# Impact of Simulated Artifacts on the Classification Performance of Apical Views in Transthoracic Echocardiography Using Convolutional Neural Networks

**DOI:** 10.3390/bioengineering13050522

**Published:** 2026-04-30

**Authors:** Gabriela Bernadeta Orzeł-Łomozik, Łukasz Łomozik, Maciej Podolski, Martyna Rożek, Kalina Światlak, Weronika Radwan, Zuzanna Przybylska, Paulina Michalska, Maciej Pruski, Katarzyna Mizia-Stec

**Affiliations:** 1 1st Department of Cardiology, Medical University of Silesia, 40-055 Katowice, Poland; maciekmcee@onet.eu (M.P.); martynarozek0704@gmail.com (M.R.); swiatlakkalina@gmail.com (K.Ś.); s86139@365.sum.edu.pl (W.R.); s86122@365.sum.edu.pl (Z.P.); pmichalska000@gmail.com (P.M.); kmiziastec@gmail.com (K.M.-S.); 2 Faculty of Mechanical Engineering, Silesian University of Technology, 44-100 Gliwice, Poland; llomozik@polsl.pl; 3 Department of Cardiology, Polish-American Heart Clinics, 43-100 Tychy, Poland; maciejpruski87@gmail.com

**Keywords:** AI, transthoracic echocardiography, CNN, image quality, motion blur, acoustic shadowing, speckle noise

## Abstract

**Background:** In recent years, artificial intelligence (AI) methods, including deep convolutional neural networks (CNNs), have gained increasing importance in supporting the automated analysis of echocardiograms. The aim of this study was to evaluate the impact of selected image artifacts—motion blur, acoustic shadowing, and speckle noise—on the performance of automatic classification of standard transthoracic echocardiographic (TTE) views using deep learning models. **Methods:** The analysis included 217 TTE video clips (2170 frames) covering apical views: two-chamber (A2C), three-chamber (A3C), four-chamber (A4C), and five-chamber (A5C). Two convolutional neural network architectures—ResNet-18 and ResNet-34—were applied, initialized with weights pretrained on the ImageNet dataset (transfer learning). In a limited comparative scope, EfficientNet-B0, a ViT model used as a frozen feature extractor combined with Logistic Regression, and a classical HOG + SVM model, were also included as reference methods. Classification performance was evaluated under conditions of controlled image degradation caused by motion blur, acoustic shadowing, and speckle noise. **Results:** All analyzed artifacts reduced classification performance, although the magnitude of this effect depended on artifact type. Speckle noise proved to be the most destructive, causing performance collapse across all evaluated methods at high severity. Motion blur and acoustic shadowing produced more differentiated degradation profiles. The ResNet models achieved the highest performance under reference conditions; however, after degradation, the ranking of models was no longer stable. In the comparative analysis, HOG + SVM showed the smallest relative performance loss under motion blur and the highest balanced accuracy under severe acoustic shadowing, whereas severe speckle remained critical for all models. **Conclusions:** Image quality degradation significantly impairs TTE view classification performance, and evaluation based solely on reference-quality images does not fully reflect model robustness to artifacts. These findings indicate the need to complement standard model evaluation with a structured robustness analysis under degraded imaging conditions and highlight the importance of training and validation settings that better reflect real clinical practice.

## 1. Introduction

What is known

Automatic identification of standard echocardiographic views in transthoracic echocardiography (TTE) constitutes a prerequisite for reliable quantitative analysis, both from a clinical perspective—enabling standardized acquisition and reporting—and from an algorithmic perspective, as it conditions the activation of view-dependent models for segmentation, parameter estimation, and disease detection. In clinical practice, the guidelines of the American Society of Echocardiography [1] define a set of key projections and recommendations for their acquisition during comprehensive examinations, emphasizing that a “correct view” represents the fundamental organizational unit for echocardiographic interpretation [1]. Additionally, the recommendations of the British Society of Echocardiography [2] highlight the importance of a structured acquisition protocol as a prerequisite for measurement comparability and examination completeness [2,3]. The literature demonstrates that convolutional neural network (CNN)-based view classification can achieve performance approaching expert-level accuracy when test data closely match the training data distribution. Studies addressing the recognition of multiple standard TTE views have reported very high accuracy (on the order of ~98% for video clips), with similarly strong performance maintained at the single-frame level, indicating that echocardiographic view classification is practically feasible and clinically usable [3]. The feasibility of real-time operation has also been demonstrated, with reported accuracies of approximately 98–99% and inference times on the order of a few milliseconds per frame, which is critical for integrating such models directly into the acquisition workflow [4]. Subsequent studies further confirm that view classification is widely regarded as a foundational step enabling downstream stages of automated echocardiogram interpretation [4,5]. At the same time, real-world clinical conditions introduce substantial heterogeneity in image quality. Ultrasonography is a highly operator-dependent modality and is inherently susceptible to artifacts arising from wave-propagation physics, acquisition geometry, and acoustic window limitations. Reviews of artifacts in echocardiography and ultrasonography emphasize phenomena such as reverberations, lobe artifacts (including side-lobe artifacts), and acoustic shadowing, both in terms of their potential to cause misinterpretation and their diagnostic limitations [6,7,8]. In the context of apical views, clutter phenomena—often associated with reverberation mechanisms—have also been described as degrading structural visibility and overall diagnostic value [5,9]. In recent years, increasing attention has been devoted to two additional aspects critical for the safe deployment of models in clinical practice: (i) the detection of unknown, out-of-distribution (OOD), or non-classifiable/poor-quality cases, and (ii) automated image quality assessment. For example, approaches have been proposed that combine routine view classification with the recognition of unknown views, including low-quality cases, as well as with quality regression or scoring, achieving moderately high performance for the joint objective and rank-level agreement with expert assessments of view quality [10]. Independently, quality assessment models have been introduced in echocardiography to support more reproducible measurements (e.g., in strain analysis) and to reduce variability [11]. More recently, large-scale multitask solutions combining view classification and quality assessment, trained on extensive clinical datasets, have also been reported [6,12]. In the context of deep learning architectures, convolutional neural networks (CNNs) remain the dominant approach in medical image analysis, demonstrating strong performance, particularly in settings with limited data availability. Transformer-based architectures, such as Vision Transformer (ViT), have been introduced to capture global dependencies within images and have shown promising results in various medical imaging tasks; however, their performance often depends on large-scale datasets and pretraining strategies [13]. Consequently, in many practical clinical scenarios characterized by limited data, CNN-based models remain a robust baseline, while transformer-based approaches are frequently used in a complementary or comparative capacity [14]. From the broader perspective of deep learning, it is well established that standard CNNs can be highly sensitive to so-called common corruptions (e.g., blur, noise, or digital artifacts) when such degradations are not adequately represented during training and when their severity increases. In the computer vision literature, formalized robustness benchmarks—most notably ImageNet-C and ImageNet-P—have been introduced to standardize the evaluation across corruption types and severity levels and to facilitate model comparison under distribution shift [15]. In particular, blur and noise have repeatedly been identified as degradations leading to substantial performance drops in CNN-based recognition tasks [16,17]. Furthermore, it has been shown that models trained on natural images tend to rely heavily on textural cues, which can further compromise prediction stability when texture becomes unreliable due to noise or blurring [7,18]. In ultrasonography, speckle is a signal-dependent phenomenon, often modeled as multiplicative noise, and is known to hinder segmentation and image analysis tasks, motivating both speckle reduction techniques and physics-aware reconstruction and augmentation strategies [19,20]. Reviews of deep learning applications in ultrasonography consistently emphasize that robustness to quality degradations is a prerequisite for clinical trustworthiness [21,22]. Moreover, studies on augmentation strategies for ultrasound indicate that augmentation practices are often inconsistent, and although ultrasound-specific augmentations (e.g., acoustic shadows, speckle maps, or time-gain compensation effects) are being introduced, they require systematic validation across tasks and settings [8,23,24,25].

What is missing

Despite the high view-classification performance reported in the literature, there is often a lack of quantitative, transferable characterization of how specific image quality degradations affect recognition metrics in a manner that allows comparison across studies. In particular, many works demonstrate strong performance on data closely resembling the training distribution but do not report curves of the form “artifact type → severity level → degradation in accuracy/balanced accuracy/AUC,” leaving the safe operating boundary of models under degradation largely undefined [3,4,9,15].

A second limitation is the absence of a standardized robustness benchmark specific to echocardiography, analogous in spirit to ImageNet-C, that would map common corruptions to realistic ultrasound/TTE degradations (e.g., speckle with controlled intensity, shadow-like signal dropout, motion blur, contrast or resolution degradation, and digital artifacts). While mature benchmarks in computer vision have enabled result comparability and aggregate robustness metrics, TTE studies still predominantly report performance under single test conditions or heterogeneous degradation setups [10,15,16].

A third gap concerns the relationship between image quality enhancement and improvements in semantic tasks. In the ultrasonography literature, numerous studies focus on reconstruction or denoising metrics (e.g., PSNR, SSIM, or contrast-to-noise measures); however, evidence that improvements in these metrics translate into consistent gains in tasks such as view classification or pathology detection is often limited or highly dependent on the specific architecture and dataset [20,22]. In practice, this implies that a “better image” in terms of pixel-level metrics does not necessarily correspond to a “safer decision” in terms of clinically relevant error [11].

A fourth issue is operational in nature. Despite the growing number of methods for quality assessment and unknown/OOD detection in echocardiography, there is a lack of a unified framework that explicitly links artifacts (as causes), image quality (as a state), classifier decisions (as outcomes), and the resulting structure of decision errors (e.g., class-to-unknown transitions, confidence drops, entropy increases, or calibration shifts) [10,11,12,15]. Existing studies demonstrate the feasibility of recognizing unknown cases and assessing quality, but they do not yet establish a standardized robustness testing paradigm in which these components are evaluated in a clearly comparable manner across studies and implementations [10,12,26,27]. Finally, although clinically relevant deep learning pipelines in echocardiography exist—integrating view identification, segmentation, and parameter estimation such as ejection fraction—the relationship between quality degradation or view misclassification and the reliability of final clinical indicators, as well as the associated diagnostic risk, remains poorly quantified. In practice, many solutions implicitly assume correct view selection and sufficient input quality, effectively shifting risk to upstream quality control and data selection stages [15,28,29].

What this study does

The present study is positioned within the domain of robustness evaluation for image quality degradation in standard TTE view classification. A structured protocol was applied to assess the effect of image degradations on classification metrics using an “artifact type × severity level → degradation curve” paradigm, analogous to benchmark approaches developed in computer vision for common corruptions [15]. The selected degradations included artifacts that are clinically typical and/or fundamental to ultrasonography—namely speckle noise, acoustic shadowing/signal dropout, and motion blur—consistent with reviews of ultrasound and echocardiographic artifacts and their underlying mechanisms [6,7,8,9,16,19]. At the experimental level, the study examined how controlled, progressively increasing degradations affect the recognition of routine apical TTE views by comparing two CNN architectures of different depth (ResNet-18 and ResNet-34) and, in a limited comparative scope, EfficientNet-B0, a ViT model used as a frozen feature extractor combined with Logistic Regression, as well as the classical HOG + SVM baseline. The purpose of this comparison was to assess whether the observed degradation profiles remain stable across different image-representation families, rather than to identify a single best-performing classifier. In this sense, the study contributes not only a quantitative description of how artifacts affect apical TTE view classification, but also a structured analysis of model robustness under degraded imaging conditions.

## 2. Materials and Methods

### 2.1. Data Characteristics

The experiment was conducted using a dataset of transthoracic echocardiographic images comprising standard apical views: two-chamber, three-chamber, four-chamber, and five-chamber. Each observation was represented as a short video clip composed of several to a dozen consecutive frames; on average, 10 frames per clip were used, yielding a total of 2170 frames.

In total, 217 video clips (2170 frames) were collected and subsequently divided into training, validation and test sets. A detailed distribution of frames assigned to each cardiac projection class is presented in Table 1.

The transthoracic echocardiographic images used in our study were acquired at the 1st Department of Cardiology, Faculty of Medical Sciences in Katowice, Medical University of Silesia, Katowice, by physicians specialized in performing transthoracic echocardiography. The operators’ competencies are certified by the Echocardiography Working Group of the Polish Cardiac Society. Moreover, each examination was independently validated by a second expert echocardiographer in order to minimize the risk of image misinterpretation and to ensure the high reliability of the material used for analysis.

The data collection methodology focused on the analysis of transthoracic echocardiographic images obtained from adult patients across all adult age groups. The patients underwent transthoracic echocardiography during hospitalization in the 1st Department of Cardiology. The study was approved by the Bioethics Committee of the Medical University of Silesia in Katowice, approval number: BNW/NWW/0052/KB1/105/23/24.

### 2.2. Data Processing Methods

Echocardiographic images were subjected to a multistage preprocessing pipeline comprising frame extraction from multi-frame studies, standardization of image quality, and preparation of the data for use as input to the classification model. All frames were represented as RGB images rescaled to a uniform resolution of 256 × 256 pixels and normalized using the statistical parameters of the ImageNet dataset. Data augmentation included random rotations (±10°) and random horizontal flips. The model operated on individual frames and did not incorporate temporal information from the original video sequences.

The division into training and test sets was performed at the level of complete echocardiographic sequences, ensuring that all frames originating from a given recording were assigned to the same subset. The split was stratified with respect to the view class distribution at the sequence level and controlled using a fixed random seed. The test set comprised 20% of all sequences. A validation subset was created by applying a stratified random split (20%) to the training labels using a fixed random seed. This split was performed at the frame level, meaning that frames originating from the same echocardiographic video could be present in both the training and validation subsets. The validation set was used exclusively for hyperparameter optimization and early stopping and was not treated as an independent evaluation dataset.

Frames extracted from the same echocardiographic sequence are inherently temporally correlated and therefore cannot be considered statistically independent samples. To mitigate data leakage between training and evaluation, all frames originating from a given sequence were assigned exclusively to a single dataset split (training or test). Nevertheless, residual intra-sequence correlation remains within each subset and is considered in the interpretation of the results.

### 2.3. Neural Network Architectures

The analysis employed ResNet-18 and ResNet-34 architectures initialized with weights pretrained on the ImageNet-1K dataset. The convolutional structure of both models remained unchanged relative to their canonical reference implementations. Adaptation to the task of echocardiographic view classification involved replacing the original fully connected layer with a module consisting of a Dropout layer (*p* = 0.3) followed by a linear output layer with the number of neurons corresponding to the number of classes.

Both models processed images as individual frames with a fixed input size and did not incorporate information related to cardiac motion. To characterize the computational demands of the architectures, the number of trainable parameters, the number of floating-point operations (FLOPs), and the average inference time for a single 256 × 256-pixel frame were estimated. Measurements were performed on a CPU under identical conditions for both models. The results are summarized in Table 2.

ResNet-34 exhibits nearly twice as many parameters and a higher computational complexity compared with ResNet-18, which translates into a longer inference time.

To relate the results to alternative approaches reported in the literature [13,14,30,31,32,33,34], three additional reference models were included in the analysis with a limited comparative role. The first was EfficientNet-B0, selected as a representative convolutional architecture characterized by high parameter efficiency. The second was a Vision Transformer (ViT), included as a representative of transformer-based architecture. The third was a classical HOG + SVM model, in which the Histogram of Oriented Gradients (HOG) descriptor was used for image representation, and classification was performed with a Support Vector Machine (SVM). The inclusion of this method was motivated by earlier studies on echocardiographic view classification that employed classical approaches based on handcrafted features and SVM classifiers, including HOG, bag-of-words (BoW) [34,35], and related descriptors. Moreover, for apical views, successful non-deep-learning approaches based on spatio-temporal features and dictionary learning [33] have also been reported. Due to the limited size of the dataset and the high data requirements of transformer models reported in the literature, the ViT model was not trained end-to-end and was not fully fine-tuned. Instead, it was used in a simplified configuration as a frozen feature extractor initialized with ImageNet-1K-pretrained weights, and the resulting feature representations were passed to a Logistic Regression classifier. To ensure comparability across model families, all reference methods were evaluated using the same test set and the same image degradation protocol. For all models, three artifact types—motion blur, acoustic shadowing, and speckle noise—were analyzed at the same severity levels. Balanced accuracy was used as the primary comparative metric. This comparison was limited in scope and was intended only as a contextual reference to alternative approaches reported in the literature.

### 2.4. Training Procedure and Hyperparameter Optimization

Model training was performed using the AdamW optimizer (Adaptive Moment Estimation with decoupled Weight Decay) with two learning-rate groups: 1 × 10^−4^ for all convolutional layers and 5 × 10^−4^ for the classification head. Regularization was introduced via a weight-decay coefficient of 1 × 10^−4^. To mitigate overfitting, early stopping was applied with a patience of five epochs. Model performance during training was assessed using overall classification accuracy and class-wise balanced metrics, including the F1-score. Due to class imbalance, class-weighted loss functions were employed.

Hyperparameter optimization was conducted using the Optuna library with the Tree-structured Parzen Estimator algorithm. The search space included learning rates ranging from 1 × 10^−5^ to 1 × 10^−3^, weight-decay values between 1 × 10^−6^ and 1 × 10^−3^ (both on logarithmic scales), and a binary choice between freezing the feature extractor and full fine-tuning of all layers. Configurations were evaluated on a validation set comprising 20% of the training data, with each trial trained for five epochs using a batch size of 32. The configuration achieving the highest validation accuracy corresponded to full fine-tuning, which was subsequently adopted in the main experiments.

The optimization procedure described above concerned the primary CNN models used in the main analysis. EfficientNet-B0 was trained analogously to the ResNet architectures, using ImageNet-1K-pretrained weights followed by fine-tuning on the study dataset. In contrast, the ViT model was used only as a limited reference model: the backbone remained frozen, and classification was performed using a Logistic Regression classifier trained on feature representations extracted by the pretrained model. For the Logistic Regression classifier, the regularization parameter was selected on the validation set.

To further mitigate overfitting, model selection was based on validation loss minimization with early stopping (patience = 5 epochs). The validation set was used exclusively for hyperparameter tuning and monitoring training convergence, while the independent test set served solely for final performance assessment. Additional regularization strategies included, like weight decay, label smoothing (0.05), weighted sampling to address class imbalance, and cosine learning-rate scheduling. Overfitting was monitored through analysis of training–validation loss divergence, and final model performance was reported only after training completion on the held-out test set. Cross-validation (k-fold) was not applied. Instead, model generalization was evaluated using a strictly separated independent test set, reducing the risk of optimistic bias in reported results.

A concise summary of the final training configuration used in the main experiments is provided in Table 3.

The configuration of the additional reference models used for comparison is summarized in Table 4.

In the HOG + SVM model, input images were processed in grayscale, and HOG features were computed after standardizing image size; details of this comparative analysis are provided in Section 3.6.

### 2.5. Grad-CAM Explainability Analysis

To investigate the internal decision-making process of the trained convolutional neural networks, Gradient-weighted Class Activation Mapping (Grad-CAM) was used to visualize image regions contributing most strongly to model predictions. Grad-CAM generates localization maps by computing gradients of the predicted class score with respect to the feature maps of the final convolutional layer, allowing identification of spatial regions that influence the model’s classification decision. Grad-CAM activation maps were generated for representative correctly classified images as well as for images degraded using simulated artifacts (motion blur, speckle noise, and acoustic shadowing). For each case, the activation map was overlaid on the original ultrasound frame to highlight the regions receiving the highest model attention. This analysis was used to qualitatively assess whether the CNN models relied on anatomically meaningful structures when making predictions and to investigate how image degradations altered the spatial distribution of model attention.

### 2.6. Statistical Analysis

The classifier operates on individual echocardiographic frames; therefore, the primary performance metrics (accuracy, precision, recall, F1-score, and AUC) were computed at the frame level on the independent test set. However, because frames originating from the same echocardiographic clip are temporally correlated and cannot be considered fully independent observations, uncertainty estimates were obtained using a clustered bootstrap procedure at the sequence level. In each bootstrap iteration, echocardiographic sequences were sampled with replacement, and all frames belonging to the sampled sequences were included in the resampled test set. Confidence intervals were then calculated from the empirical bootstrap distribution. Additionally, the comparative analysis of reference models presented in Section 3.6 was performed at the sequence level. For this purpose, frame-level predictions were aggregated within each sequence by averaging class probabilities, and the final sequence label was assigned as the class with the highest mean probability. This approach was adopted to reduce the influence of intra-sequence frame correlation and to provide a more conservative comparison across model families.

Model performance was evaluated using frame-level accuracy, balanced accuracy, precision, recall, F1-score, and area under the receiver operating characteristic curve (AUC). Balanced accuracy was defined as the arithmetic mean of recall values across all classes and was used to account for the class imbalance present in the dataset.

For multi-class evaluation, macro-averaged metrics were reported, defined as the unweighted arithmetic mean of the class-specific metrics, thereby assigning equal importance to each projection class irrespective of class frequency. Macro-AUC was computed using a one-versus-rest (OvR) strategy, where for each class a binary ROC curve was constructed against all remaining classes, and the final value was obtained as the arithmetic mean across classes.

For Table 3 and Table 4, 95% confidence intervals for performance metrics were estimated using a non-parametric clustered percentile bootstrap procedure. Specifically, 1000 bootstrap samples were generated by sampling echocardiographic sequences with replacement from the test set, while retaining all frames belonging to each sampled sequence. Confidence intervals were defined as the 2.5th and 97.5th percentiles of the resulting bootstrap distribution (α = 0.05).

Bootstrap resampling was performed with a fixed random seed to ensure reproducibility. These confidence intervals reflect the variability of performance estimates on the test dataset and were not used for hypothesis testing.

The effect of image artifacts was quantified by comparing frame-level performance metrics under artifact conditions with the corresponding baseline (non-degraded) performance. To preserve the dependency structure of the data, confidence intervals for Δ were estimated using paired clustered bootstrap resampling at the sequence level. In each bootstrap iteration, echocardiographic sequences were sampled with replacement, and both baseline and degraded metrics were recomputed on the same resampled set (Table 5, Table 6, Table 7, Table 8, Table 9 and Table 10). Statistical significance of degradation effects was assessed using bootstrap-based confidence intervals and empirical bootstrap *p*-values. To account for multiple comparisons across artifact intensity levels, *p*-values were adjusted using the Holm step-down procedure.

An analogous sequence-level bootstrap procedure was also applied for between-model comparisons under the same degradation conditions.

To quantify the impact of training dataset size on classification performance, a learning curve analysis was conducted. The CNN models were trained using 25%, 50%, 75%, and 100% of the available training data, while keeping the validation and test sets unchanged. For each training fraction, model training was repeated using different random seeds to account for the stochastic nature of neural network optimization.

Model performance was evaluated on the same independent test set using accuracy, balanced accuracy, macro-F1 score, and macro-AUC. This procedure enabled direct comparison of classification performance across different training dataset sizes.

### 2.7. Simulation of Image Quality Degradation

To evaluate the robustness of the classifier to echocardiographic artifacts, three types of distortions were applied to the test images.

#### 2.7.1. Motion Blur

Motion blur was simulated by convolving the image with a linear blur kernel of a specified length, reproducing the effect of camera movement. The artifact was introduced by performing a convolution of the image I(x,y) with a linear blur kernel K(x,y) of a given length L (kernel size). The resulting image can be expressed as:

(1) $$I_{blur}\left(x,y\right)=\left(I\cdot K\right)\left(x,y\right)=\sum_{u=-m}^{m}\sum_{v=-n}^{n}I\left(x-u,y-v\right)K(u,v),$$

where K(u,v) denotes the blur filter of dimensions m×n.

In the experiment, unidirectional horizontal blurring was applied, corresponding to the situation of probe displacement along one axis during acquisition. In this case, the kernel took the form of a vector:

(2) $$K=\;\frac{1}{L}\left[1\;1\;1\;\ldots 1\right],$$

Here, all elements take the value of $\frac{1}{L}$, while the remaining values outside the horizontal direction are set to 0.

**Figure 1:**
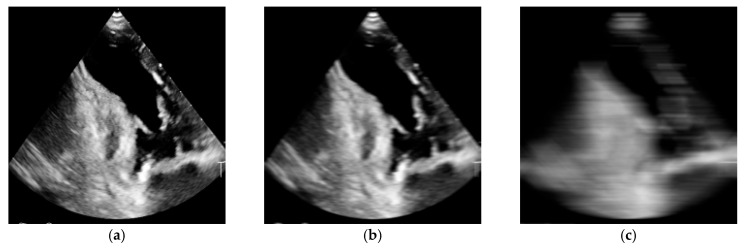
Example of simulated motion blur artifact in a transthoracic echocardiographic image. From left: (**a**) reference image without distortion (kernel size = 0), (**b**) image with mild blurring (kernel size = 5), (**c**) image with severe blurring (kernel size = 20).

The intensity of the artifact was controlled by the parameter L—values ranging from 0 (no blur) to 20 (severe blurring), tested with an increment of 2.5. A 5-pixel kernel corresponded to minor probe movement (minimal blurring), whereas a 20-pixel kernel produced pronounced streaking and the loss of most image details (Figure 1).

#### 2.7.2. Acoustic Shadowing

The acoustic shadow artifact was simulated by superimposing a conical mask on the image, originating from the upper edge and extending downward toward the apex of the echocardiographic field of view. This approach reflects the real clinical situation in which structures with high ultrasound attenuation (e.g., ribs, prostheses, implants) block acoustic wave propagation, resulting in a signal-free region behind the obstruction.

The mask was defined as a conical area with an opening angle θ, whose base width corresponded to the parameter shadow width. This parameter controlled the extent of the occluded region, ranging from 0 (no shadow; reference image) to 0.7 (image with a wide shadow).

**Figure 2:**
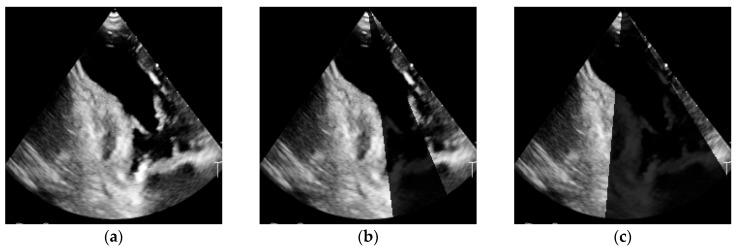
Example of simulated acoustic shadowing artifact in a transthoracic echocardiographic image. From left: (**a**) reference image without distortion (shadow width = 0), (**b**) image with a narrow shadow (shadow width = 0.3), and (**c**) image with a wide shadow (shadow width = 0.7).

Mathematically, the transformation of the image I(x,y) can be expressed as:

(3) $$I_{shadow}\left(x,y\right)=\{\epsilon \;dla\left(x,y\right)\in \Omega_{shadow}\;I\left(x,y\right)\;dla\left(x,y\right)\notin \Omega_{shadow}\;$$

where $\Omega_{shadow}$ denotes the conical mask region, and ϵ is a small random value added to simulate background noise (typically $\epsilon \;∼N\left(0,\;\sigma^{2}\right),\;$with σ corresponding to a few percent of the mean image brightness). As a result, the shadow was not entirely uniform but exhibited a heterogeneous character, more closely resembling artifacts observed in clinical practice (Figure 2).

#### 2.7.3. Speckle Noise

Speckle noise is a characteristic artifact of ultrasound imaging, resulting from the interference of acoustic waves reflected by numerous small inhomogeneities within tissues. It produces a fine-grained, irregular texture uniformly distributed across the entire image, which hinders the detection of anatomical structures.

In the experiment, a multiplicative speckle noise model was applied, defined by the following equation:

(4) $$I_{noisy}\left(x,y\right)=I\left(x,y\right)+I\left(x,y\right)\cdot N\left(0,\sigma^{2}\right),$$

For simplification of the analysis, a parameter termed noise amount was introduced, corresponding to the standard deviation σ relative to the mean image brightness. This parameter ranged from 0 (no additional noise, reference image) to approximately σ ≈ 3, which generated images with a pronounced “snow-like” texture, hindering the recognition of anatomical details.

**Figure 3:**
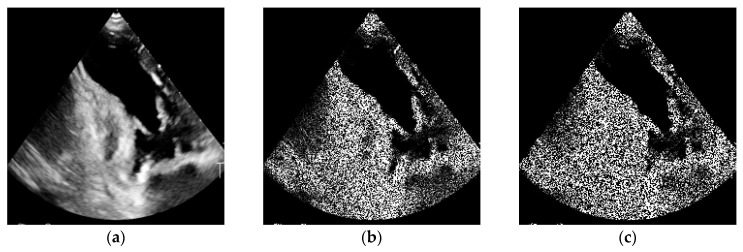
Example of simulated speckle noise artifact in a transthoracic echocardiographic image. From left: (**a**) reference image without distortion (noise amount = 0), (**b**) image with a moderate noise level (noise amount = 1), (**c**) image with a high noise level (noise amount = 3). With increasing values of the noise amount parameter, the granular speckle pattern becomes more pronounced, hindering the interpretation of the echocardiogram.

Owing to the multiplicative nature of this model, the introduced noise exhibited varying intensity depending on the local brightness values of the image, thereby producing a realistic visual effect typical of echocardiography Figure 3.

#### 2.7.4. Rationale for Parameter Range Selection in Image Degradation Simulations

The parameter ranges used for the simulated degradations—motion blur, speckle noise, and acoustic shadowing—were selected to encompass the spectrum of artifact intensities typically encountered in transthoracic echocardiography, while maintaining numerical stability of the transformations and ensuring clinical interpretability of the resulting images. The selection was informed by an initial qualitative assessment of real-world TTE examinations and by pilot simulations aimed at identifying distortion levels associated with the loss of anatomical readability.

The upper limit of speckle noise intensity (σ ≈ 0.20) was chosen based on observations that higher values result in a rapid increase in granular texture and a disappearance of cardiac structural contours, producing images that appear unnatural or clinically unusable. Pilot simulations demonstrated that for σ > 0.20, nearly all tissue boundaries become obscured across the entire field of view, a pattern not representative of typical clinical conditions.

Similarly, the range of acoustic shadow width was restricted to 0–0.7, where a value of 0.7 corresponds to a shadow covering approximately 70% of the image width. Wider shadows obscure nearly all anatomical structures, generating artifacts that are rarely encountered in practice except in cases of severely impaired acoustic windows or improper transducer positioning. The adopted range therefore captures scenarios from mild, partially obscuring shadows—commonly seen in suboptimal-quality studies—to severe but still clinically plausible shadowing.

The plausibility of the simulated artifacts was assessed qualitatively rather than quantitatively. Because no separate reference dataset of clinically annotated artifact-containing images was available, parameter ranges were selected using a clinically informed expert review procedure. Specifically, the generated images were visually inspected by experienced echocardiography readers to determine whether the simulated degradations remained consistent with artifact patterns encountered in routine TTE practice and whether key anatomical structures were still partially recognizable at lower and moderate severity levels. Parameter values that produced visually unrealistic images or near-complete loss of anatomical interpretability were excluded from the final experimental range.

#### 2.7.5. Experimental Procedure

**Figure 4:**
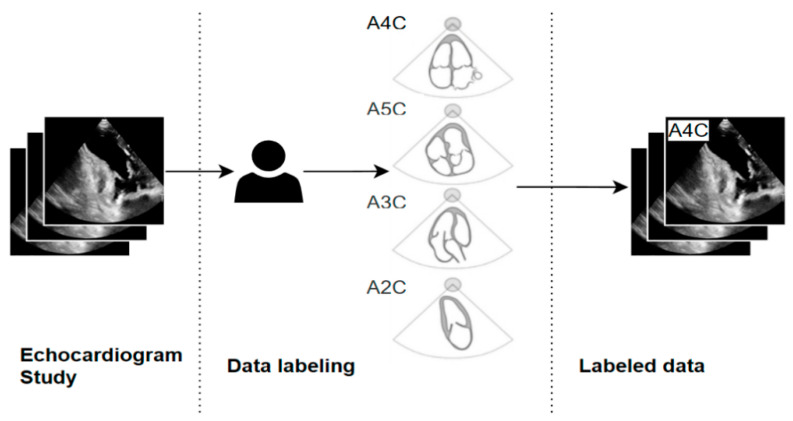
Schematic representation of the echocardiographic data labeling process. Raw TTE images were manually annotated by an expert and assigned to the corresponding cardiac views (A2C, A3C, A4C, A5C). The resulting labeled dataset constituted the basis for subsequent preprocessing and training of CNN models. (TTE—transthoracic echocardiography; A2C—apical two-chamber; A3C—apical three-chamber; A4C—apical four-chamber; A5C—apical five-chamber).

The experimental process consisted of several stages, from data preparation to the final evaluation of the classification models. Initially, manual labeling of echocardiographic data was performed (Figure 4). The collected TTE images were assigned by experts to the corresponding cardiac views (A2C, A3C, A4C, A5C), thereby creating a reference dataset. These data served as the basis for further analysis and were subjected to a preprocessing stage that included cropping and resizing to standardize image dimensions.

**Figure 5:**
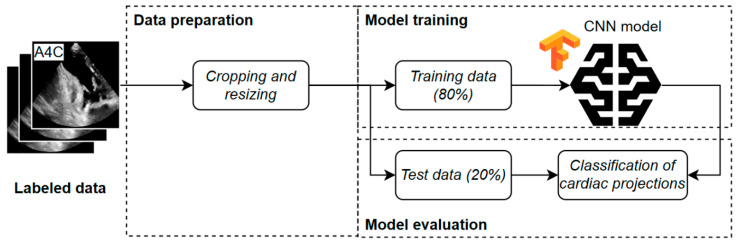
General workflow of the experiment. The labeled data were subjected to preprocessing (cropping and resizing) and subsequently divided into training and test sets. CNN models (ResNet-18, ResNet-34) were then trained to classify cardiac views. (CNN—convolutional neural network).

In the next step, the prepared images were divided into a training set (80% randomly selected data) and a test set (20% randomly selected data) (Figure 5). Deep learning was then applied to the training set using convolutional neural networks, specifically ResNet-18 and ResNet-34 architectures. The models were trained to classify echocardiographic projections and subsequently evaluated in terms of classification accuracy on the test set.

**Figure 6:**
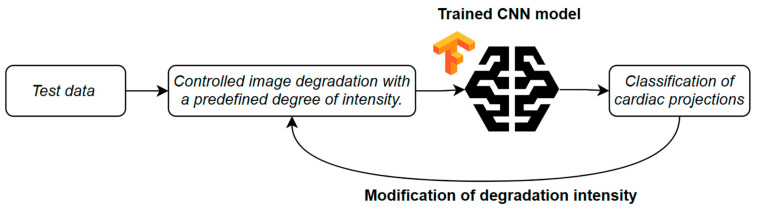
Schematic representation of the procedure for assessing CNN robustness to image degradation. Test data were subjected to controlled degradation with a defined intensity level and subsequently classified using the trained model. The process was repeated across varying degradation intensities to analyze the impact of artifacts on the classification performance of echocardiographic views. (CNN—convolutional neural network).

The core part of the experiment focused on assessing model robustness to input data degradation (Figure 6). Each test image was subjected to controlled degradation using a defined type of artifact and a specified intensity level. Multiple levels of severity were included, enabling an analysis of how image degradation affected classification performance.

For each combination of artifact type and intensity level, classification performance was assessed by calculating accuracy, precision, recall, and F1-score, with results reported separately for each cardiac projection class.

## 3. Results

Unless stated otherwise, the performance metrics presented in this section were computed at the frame level, whereas uncertainty estimates were obtained using clustered bootstrap resampling at the sequence level. An exception is Section 3.6, in which the comparison of reference models is presented primarily at the sequence level using aggregation of frame-level predictions within each sequence.

### 3.1. Classification Performance Under Reference Conditions

Table 5 and Table 6 present the principal classification metrics for the ResNet-18 and ResNet-34 models, reported separately for each echocardiographic projection class. Values in parentheses represent 95% bootstrap confidence intervals.

For ResNet-18, the highest performance was observed for class A4C (F1 = 0.902; Table 3), whereas the lowest F1-score was obtained for class A2C (0.756). Class A5C achieved perfect sensitivity (recall = 1.000) at the expense of reduced precision (0.741), indicating the absence of false-negative predictions but an increased number of false-positive assignments.

In the case of ResNet-34, the highest F1-score was likewise obtained for class A4C (0.906; Table 4), while the lowest was reported for class A5C (0.757). Compared with ResNet-18, the ResNet-34 model demonstrated higher precision for A5C (0.824) but reduced sensitivity (0.700), reflecting a more conservative decision pattern for this class.

The overall distribution of pointwise metrics (Table 5 and Table 6) indicates that both models achieve stable performance for the majority class A4C, whereas the greatest challenges arise for underrepresented classes, particularly A2C and A5C.

Because the dataset exhibits class imbalance across echocardiographic views, balanced accuracy was additionally computed to assess class-wise recognition performance. For the ResNet-18 model, balanced accuracy reached 0.867, while for ResNet-34 it was 0.811, indicating that both architectures achieved relatively consistent performance across projection classes despite differences in class frequency.

**Figure 7:**
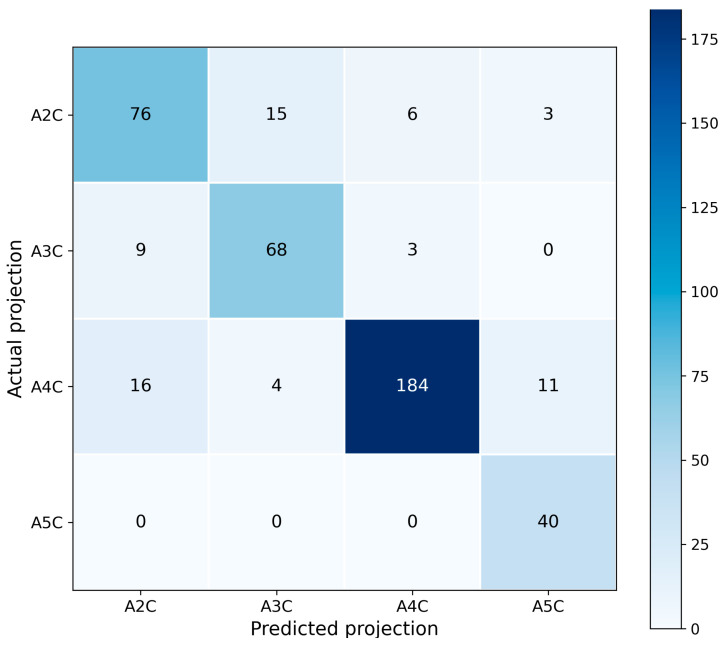
Frame-level confusion matrix for ResNet-18 on the independent test set.

**Figure 8:**
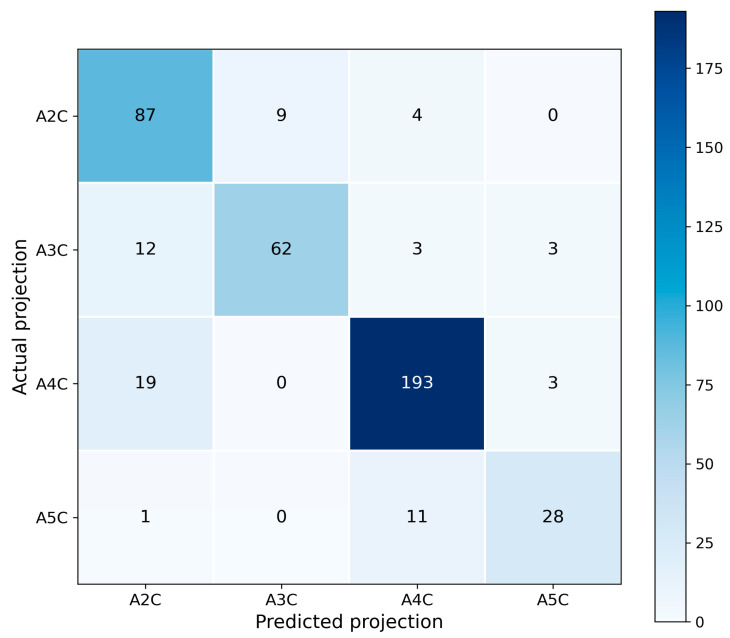
Frame-level confusion matrix for ResNet-34 on the independent test set.

Confusion matrices for ResNet-18 and ResNet-34 are presented in Figure 7 and Figure 8, respectively. In both cases, the largest number of correct predictions corresponds to class A4C, consistent with its highest support and with the strongest F1-scores reported in Table 4 and Table 5.

For class A5C, the ResNet-18 model produced no false-negative predictions but exhibited an increased number of false positives (Figure 7). In contrast, ResNet-34 generated fewer false positives but introduced false-negative errors (Figure 8). This error structure is fully consistent with the trade-off between precision and recall observed in Table 2 and Table 3.

The confusion matrices do not indicate systematic misclassification of any particular class; however, they do highlight greater ambiguity in the decision boundaries for the less frequent projections.

**Figure 9:**
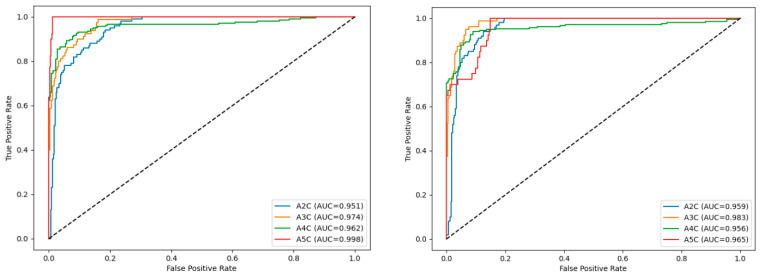
Receiver operating characteristic (ROC) curves for individual echocardiographic views obtained using the ResNet-18 model (**left**) and the ResNet-34 model (**right**). The area under the curve (AUC) values are reported separately for the A2C, A3C, A4C, and A5C classes. (A2C—apical two-chamber; A3C—apical three-chamber; A4C—apical four-chamber; A5C—apical five-chamber). The dashed diagonal line represents the performance of a random classifier, corresponding to an AUC of 0.5.

Receiver operating characteristic (ROC) curves for individual classes are shown in Figure 9 for ResNet-18 and ResNet-34, respectively.

For the ResNet-18 model, the area under the curve (AUC) values range from 0.951 (A2C) to 0.998 (A5C), demonstrating strong class separability under reference conditions. Similarly, the ResNet-34 model achieves high AUC values for all classes, exceeding 0.95 across the board, with particularly high performance for A3C and A5C views. The similarity of the ROC curve shapes between the two architectures indicates comparable ranking performance and discrimination ability. It should be noted that high AUC values do not necessarily correspond to equally high F1-scores. While AUC reflects global ranking performance across all possible classification thresholds, precision, recall, and F1-score quantify performance at a single operating point defined by the maximum-probability decision rule. Consequently, confident misclassification may still occur despite preserved AUC values, particularly under conditions that induce shifts in class probability distributions. In summary, both ResNet-18 and ResNet-34 demonstrate strong discrimination capability for apical echocardiographic views under reference conditions. Although ResNet-34 attains marginally higher AUC and accuracy values for selected classes, the overall differences relative to ResNet-18 are modest and do not indicate a qualitative change in classification behavior.

### 3.2. Effect of Training Dataset Size

**Figure 10:**
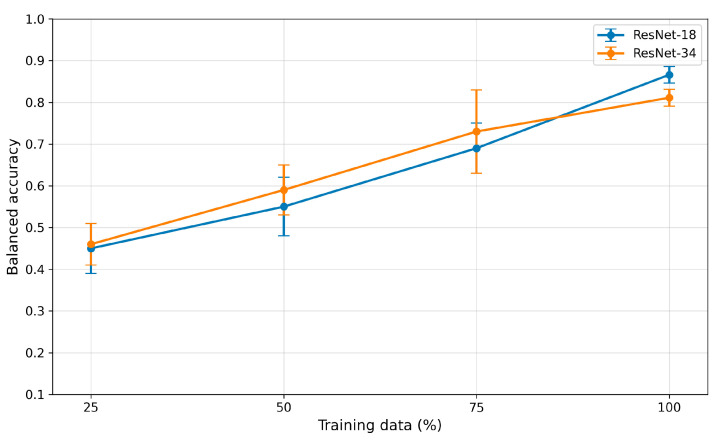
Learning curve showing the effect of training dataset size on balanced accuracy for ResNet-18 and ResNet-34 architectures.

To evaluate the impact of dataset size on model performance, a learning curve analysis was conducted using different fractions of the training data, as shown in Figure 10.

For the ResNet-18 architecture, balanced accuracy increased from approximately 0.45 when using 25% of the training data to 0.55 for 50% and 0.69 for 75%. The final performance obtained using the full training dataset reached 0.866, consistent with the reference results reported in Section 3.1. A similar trend was observed for the ResNet-34 architecture, where balanced accuracy increased from 0.46 (25% of the training data) to 0.59 (50%) and 0.73 (75%), reaching 0.811 for the fully trained model. In addition to improved performance, the variability of model results across training runs decreased with larger training datasets, indicating increased training stability.

### 3.3. Impact of Motion Blur on the Classification Performance of Cardiac Views

**Figure 11:**
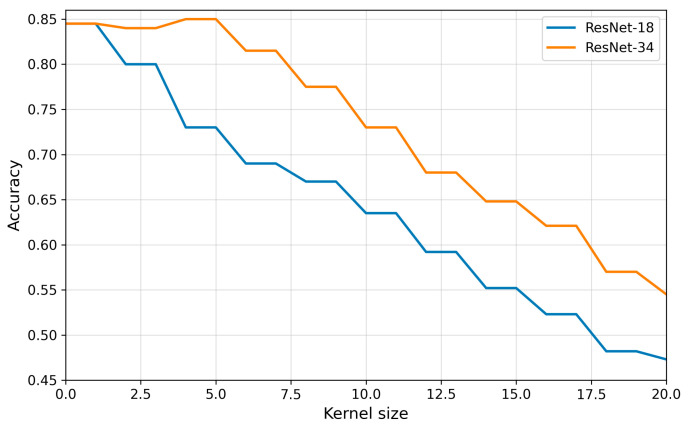
Classification accuracy as a function of motion blur kernel size for ResNet-18 and ResNet-34 models.

The analysis demonstrated that increasing the intensity of motion blur (larger kernel size) led to a systematic decline in classification accuracy (Figure 11). For artifact-free images (kernel size = 0), both architectures achieved a high performance of approximately 85%. As the blur intensified, accuracy decreased in an almost linear fashion, reaching ~50% for ResNet-18 and ~55% for ResNet-34 at kernel size = 20. This finding confirms a monotonic trend: the stronger the blur, the greater the difficulty for the models in correctly classifying cardiac views. Notably, ResNet-34 consistently outperformed ResNet-18, maintaining an advantage of about 5–7 percentage points at each blur level. This pattern can be interpreted as a consequence of the greater depth of the ResNet-34 architecture, which provides a broader range of receptive fields and supports more diverse hierarchical feature representations compared with the shallower ResNet-18. Deeper convolutional models exhibit an enhanced ability to integrate information across multiple spatial scales, thereby facilitating the extraction of features that are more robust to local image perturbations. In the context of motion blur, this implies that higher-level processing stages may preserve anatomically relevant patterns even when low-level pixel information is partially degraded.

**Figure 12:**
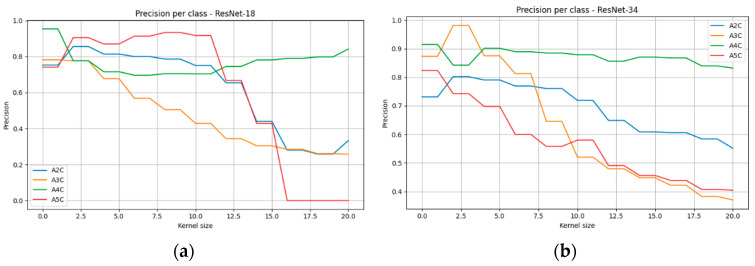

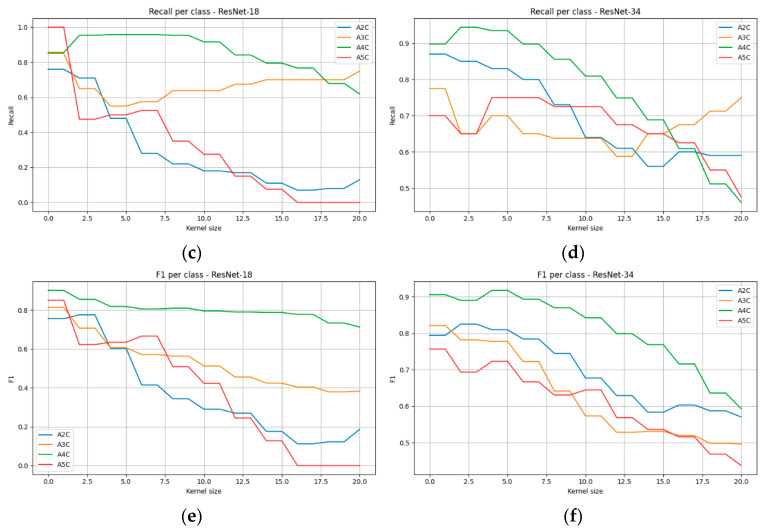
Impact of motion blur artifact on the classification performance of transthoracic echocardiographic views for ResNet-18 and ResNet-34 models. Results are presented separately for precision (**a**,**b**), recall (**c**,**d**), and F1-score (**e**,**f**).

A detailed analysis of classification metrics (precision, recall, F1-score) across individual cardiac views (Figure 12) revealed substantial variability in susceptibility to image degradation. For ResNet-18, the largest declines in precision and recall were observed for the A2C and A5C views, which, at medium and high kernel sizes, reached F1-score values close to zero, indicating an almost complete loss of discriminative ability for these projections. By contrast, the A4C view proved to be the most resistant, with precision, recall, and F1-score remaining above 0.6 even under severe blurring. Results obtained with ResNet-34 confirmed the advantage of the deeper architecture—declines in precision and recall were milder, and F1-scores across all classes remained significantly higher compared to ResNet-18. This effect was particularly evident for the A5C view, where ResNet-34 markedly reduced the number of misclassifications.

Differences in classification performance between views may be partially explained by the varying representation of classes in the training set (Table 1). The A4C view was the most frequently represented (688 frames), which facilitated more robust learning of its characteristic features and yielded greater resistance to image degradation. Conversely, the A5C view, represented least frequently (119 frames), achieved the poorest results, suggesting an insufficient number of examples to generalize patterns, especially under conditions of severe blur. The application of class weighting in the training procedure partially compensated for this imbalance, improving the overall distribution of performance metrics across classes, though it did not fully eliminate the reduced accuracy observed for underrepresented categories.

### 3.4. Impact of Acoustic Shadowing on the Classification Performance of Cardiac Views

**Figure 13:**
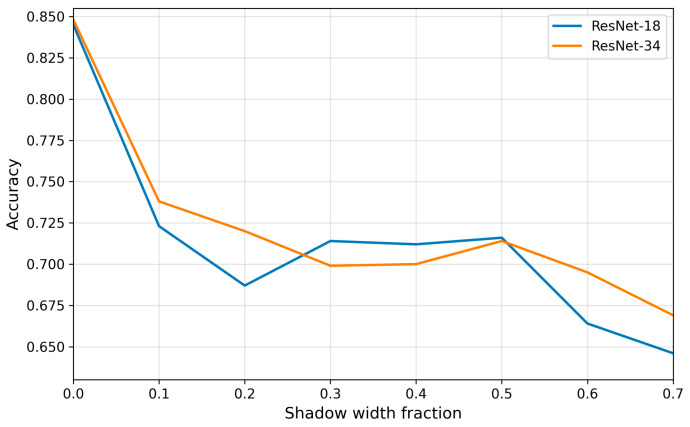
Classification accuracy of transthoracic echocardiographic views as a function of acoustic shadow width for ResNet-18 and ResNet-34 models.

The analysis showed that acoustic shadowing significantly reduced the classification performance of transthoracic echocardiographic views, with its effect evident even at small shadow widths (Figure 13). At a shadow width of 0.1, classification accuracy dropped sharply to ~72–74%. With further increases in shadow width, accuracy declined gradually, reaching <0.70 for ResNet-34 and ~0.65 for ResNet-18 under maximum shadowing. Unlike motion blur, the advantage of the deeper architecture was less pronounced—both networks responded to the loss of information in a similar manner, suggesting that acoustic shadowing masks critical image structures in a way that is difficult for deeper models to compensate.

**Figure 14:**
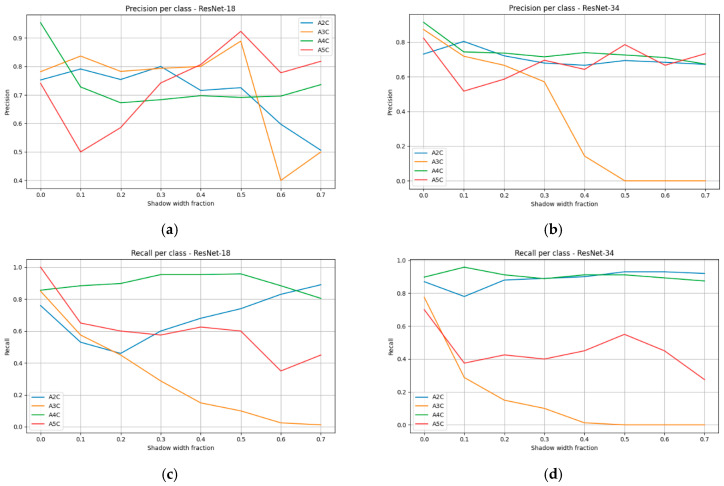

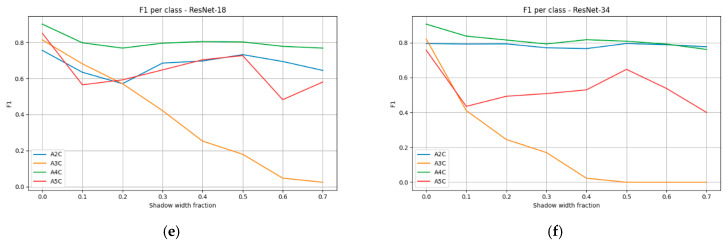
Impact of acoustic shadow width on the classification performance of transthoracic echocardiographic views for ResNet-18 and ResNet-34 models. Results are presented separately for precision (**a**,**b**), recall (**c**,**d**), and F1-score (**e**,**f**), broken down by projection classes: A2C, A3C, A4C, and A5C.

The distribution of precision, recall, and F1-score across individual projection classes (Figure 14) revealed that acoustic shadowing did not affect all views uniformly. The A4C view exhibited the greatest robustness—in both ResNet-18 and ResNet-34, F1 values remained at the level of 0.75–0.8 regardless of shadow width. This finding may be attributed to the higher number of training samples as well as the presence of distinct anatomical boundaries between cardiac chambers, which remained visible even under partial shadowing. The A2C view also demonstrated moderate stability, particularly in ResNet-34, where F1 values did not fall below 0.6.

The greatest difficulties were observed for the A3C and A5C views. For A3C, recall and F1-score decreased almost linearly, reaching values close to zero already at moderate shadowing levels (0.5–0.7). A similar trend was observed for A5C, where masking of the aortic outflow tract led to the loss of key features required for correct identification. Although ResNet-34 exhibited slightly milder declines than ResNet-18, the advantage of the deeper architecture did not resolve the problem.

### 3.5. Impact of Speckle Noise on the Classification Performance

**Figure 15:**
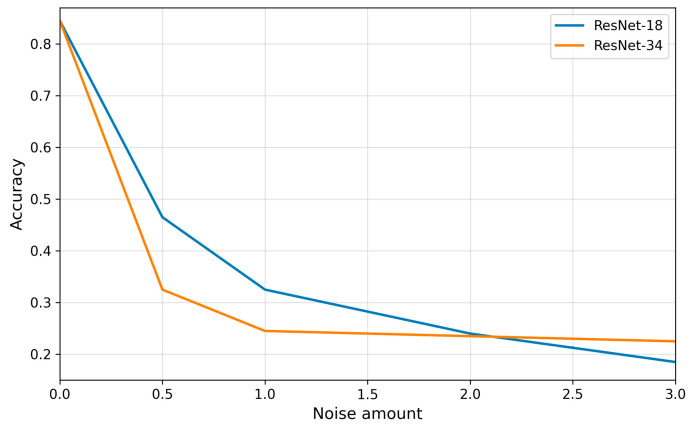
Classification accuracy as a function of speckle noise level (noise amount) for ResNet-18 and ResNet-34 models.

Speckle noise proved to be the artifact with the strongest impact on reducing the quality of transthoracic echocardiographic view classification (Figure 15). Even at low levels of interference (noise amount = 0.5), model performance declined sharply, particularly for ResNet-34, whose accuracy dropped below one-third. Further increases in noise intensity led to an almost linear deterioration of results, and at the highest parameter values, classification became largely random.

**Figure 16:**
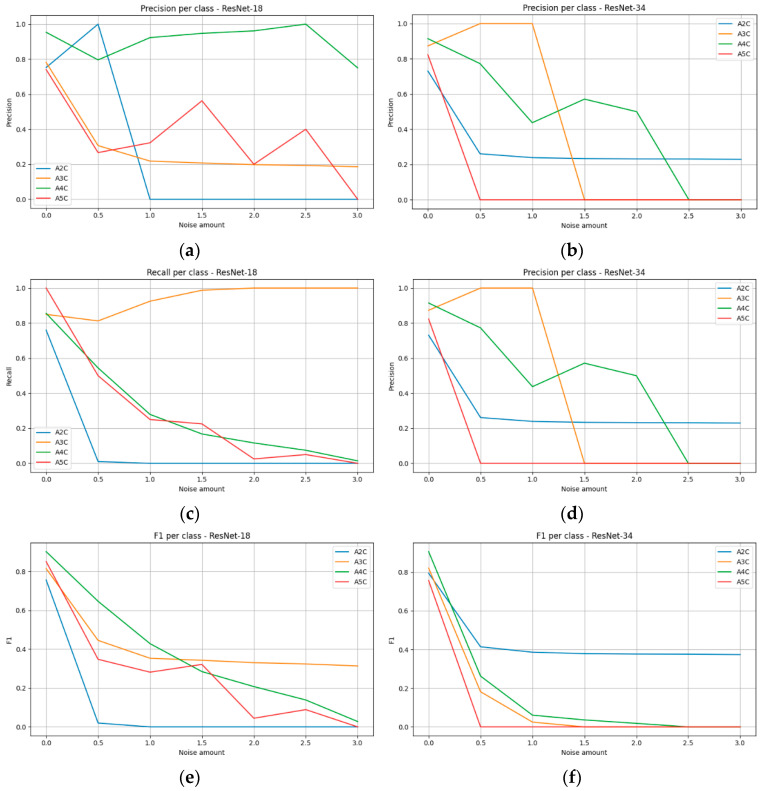
Impact of speckle noise on the classification performance of transthoracic echocardiographic (TTE) views for ResNet-18 and ResNet-34 models. Results are presented separately for precision (**a**,**b**), recall (**c**,**d**), and F1-score (**e**,**f**), broken down by projection classes: A2C, A3C, A4C, and A5C.

Analysis of individual projections (Figure 16) revealed that the A2C and A5C views were most susceptible to speckle noise, with models rapidly losing the ability to correctly recognize them. The A3C view exhibited a similar trend, although its behavior was partly dependent on the architecture. The A4C view demonstrated relatively the highest robustness, maintaining acceptable precision even at higher noise levels; however, this was accompanied by a substantial decrease in recall, resulting in a systematic decline in F1-scores.

In contrast to artifacts such as motion blur and acoustic shadowing, in the case of speckle noise, the deeper architecture did not confer a consistent advantage, as shown in Table 11 and Table 12. ResNet-34 was, in fact, more vulnerable to degradation at higher noise intensities than the shallower ResNet-18, indicating that granular interference masks subtle anatomical differences in a way that is particularly difficult to compensate for through multilayer feature representations.

### 3.6. Comparative Analysis with Additional Reference Models

To complement the main analysis with a comparison across model families, an additional comparative analysis was performed using three reference models: EfficientNet-B0, ViT (frozen) + LR, and the classical HOG + SVM model. Unlike the preceding analyses, which were presented mainly at the frame level, this comparison was reported primarily at the sequence level by aggregating frame-level predictions within each sequence using mean class probabilities. All models were evaluated on the same test set and under the same artifact simulation protocol. The primary comparative metric was sequence-level balanced accuracy.

**Figure 17:**
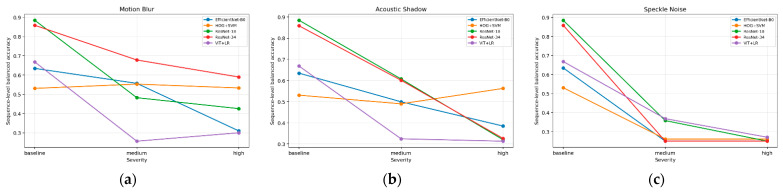
Comparison of sequence-level balanced accuracy for ResNet-18, ResNet-34, EfficientNet-B0, ViT + LR, and HOG + SVM as a function of artifact severity for: (**a**) motion blur, (**b**) acoustic shadowing, and (**c**) speckle noise.

A summary of sequence-level balanced accuracy values is presented in Table 13, whereas the changes in balanced accuracy with increasing artifact severity are shown in Figure 17.

Under reference conditions, the highest sequence-level balanced accuracy was achieved by ResNet-18 (0.885) and ResNet-34 (0.859), whereas lower values were obtained for ViT + LR (0.668), EfficientNet-B0 (0.635), and HOG + SVM (0.531). After image degradation, however, the ranking of models was no longer stable and depended on artifact type as well as on the adopted definition of robustness. For motion blur, ResNet-34 retained the highest absolute balanced accuracy at both analyzed severity levels, whereas HOG + SVM showed the smallest relative loss in performance compared with the reference condition, suggesting greater resilience of this classical representation to motion blur. This indicates that the ranking of models depended on how robustness was defined: ResNet-34 remained the best in terms of absolute performance, whereas HOG + SVM was the most stable with respect to degradation. In the case of acoustic shadowing, HOG + SVM was not the best model under reference conditions or at medium severity, yet at high severity, it achieved the highest balanced accuracy value (0.562). Speckle noise had the most destructive effect across all evaluated methods. At high severity, balanced accuracy for all models dropped to 0.250–0.270, indicating a near-complete loss of useful discriminative information.

Sequence-level bootstrap analysis showed that under high motion blur, HOG + SVM significantly outperformed EfficientNet-B0 and ViT + LR, whereas differences relative to ResNet-18 and ResNet-34 were not conclusive. Under severe acoustic shadow, HOG + SVM achieved significantly higher values than ResNet-18 and ViT + LR, whereas the comparison with ResNet-34 remained borderline after Holm correction and the difference relative to EfficientNet-B0 was not conclusive. For severe speckle noise, all models showed a significant performance decline relative to the reference condition. These findings indicate that high performance under reference conditions did not automatically translate into greater robustness to image quality degradation.

### 3.7. Failure Case Analysis Under Image Artifacts

To further elucidate the mechanisms of performance degradation, a failure case analysis was performed for sequences that were correctly classified under baseline conditions but misclassified after artifact introduction.

Table 14 presents representative failure cases from the independent test set. In all cases, the true echocardiographic view was correctly identified at baseline with high confidence (baseline true-class probability > 0.8). Following artifact introduction, a pronounced decrease in the probability assigned to the true class was observed, frequently exceeding a drop of 0.6–0.8. These probability shifts resulted in systematic misclassification rather than random prediction errors.

Motion blur primarily induced confusion between neighboring apical views, most commonly leading to misclassification of A2C or A5C sequences as A3C. This suggests that blur suppresses fine-grained anatomical features required to distinguish closely related projections. Acoustic shadowing caused misclassification toward views sharing partially visible chamber geometry, particularly when structures such as the left ventricular outflow tract were obscured. Speckle noise produced the most severe degradation, often collapsing the true-class probability to near-zero values. Notably, this effect was observed for both ResNet-18 and ResNet-34, indicating that increased network depth did not consistently improve robustness to texture-disrupting artifacts.

### 3.8. Grad-CAM Analysis of Model Attention

**Figure 18:**
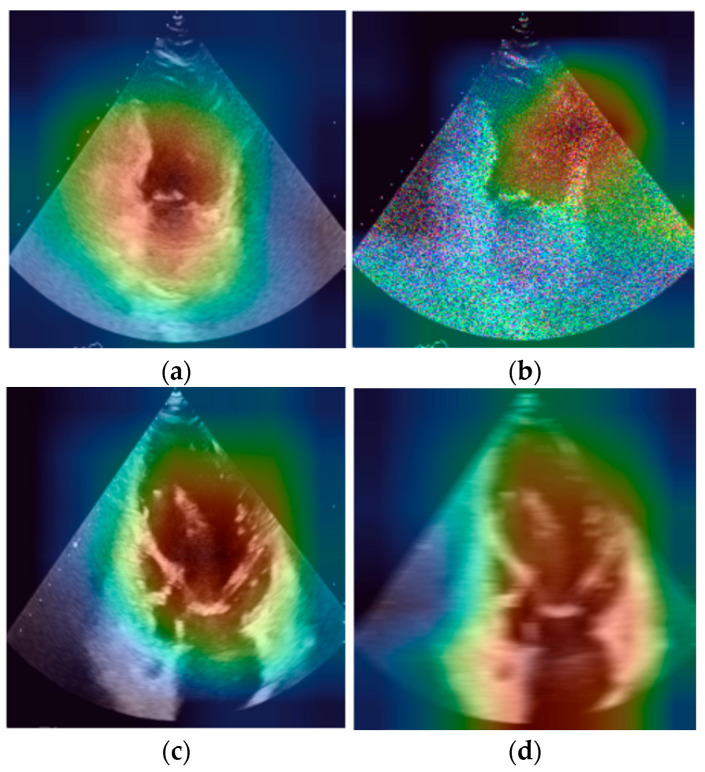

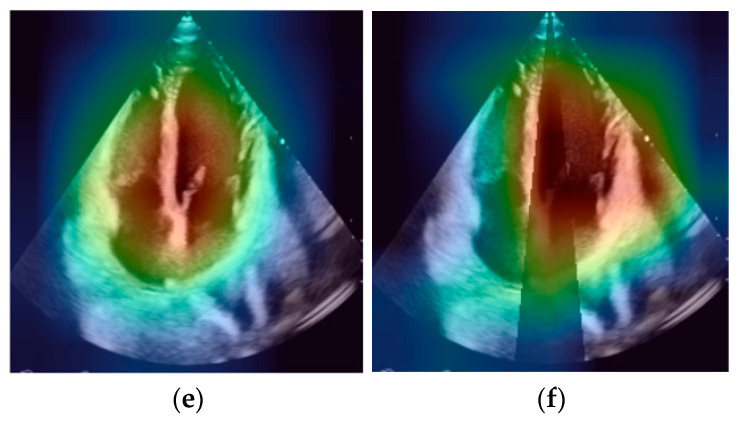
Grad-CAM visualization of CNN attention for baseline and artifact-degraded echocardiographic images. (**a**,**c**,**e**) Baseline images. (**b**,**d**,**f**) Corresponding images degraded by simulated artifacts: speckle noise (**a**,**b**), motion blur (**c**,**d**), and acoustic shadow (**e**,**f**). Warmer colors indicate image regions with higher Grad-CAM activation and stronger contribution to the model prediction, whereas cooler colors indicate regions with lower activation and weaker contribution.

To better understand how the CNN models interpret echocardiographic images, Grad-CAM activation maps were generated for selected baseline images and images degraded by simulated artifacts (Figure 18).

In the baseline examples, the activation maps are concentrated in central cardiac structures relevant for apical view recognition. After the introduction of simulated artifacts, the spatial distribution of model attention changes: speckle noise produces more diffuse activation, motion blur reduces focus on anatomical boundaries, and acoustic shadowing shifts attention away from obscured central regions.

## 4. Discussion

The experiments confirmed that degradation of echocardiographic image quality has a significant impact on the performance of cardiac view classification in TTE using convolutional neural networks. Among the analyzed artifacts, speckle noise proved to be the most destructive. Even at moderate levels of interference, a sharp decline in classification performance was observed, and under conditions of severe noise, the models lost the ability to correctly recognize projections, resulting in almost random predictions. This finding is consistent with the report by [36], who demonstrated a systematic decrease in CNN performance with increasing noise and blur levels and emphasized the need for retraining models with degraded data. Hendrycks and Dietterich [15] showed that even state-of-the-art CNNs exhibit pronounced vulnerability to common corruptions such as additive noise and signal-dependent distortions, while [37] similarly reported strong degradation of deep models under noise in real-world scenarios.

The sequence-level comparison with the classical HOG + SVM model adds an important interpretative perspective. Although HOG + SVM achieved clearly lower performance under reference conditions, it showed the smallest relative performance loss under motion blur and the highest absolute balanced accuracy under severe acoustic shadowing. This suggests that robustness to degradation depends not only on nominal model complexity but also on the type of image representation. One possible explanation is that the HOG descriptor relies primarily on global and local gradient-orientation and contour structure, whereas deep models may rely more strongly on subtler textural and contrast-related cues that are more vulnerable to blur and partial signal dropout. This interpretation is consistent with reports that CNNs are sensitive to common corruptions and often rely strongly on texture-like features [15,18], whereas their robustness can be partially improved by degradation-aware training or artifact-oriented augmentation [14]. At the same time, the HOG + SVM advantage was not uniform across all models and all pairwise comparisons; therefore, it should be interpreted as relative resilience under selected conditions rather than as a general superiority of classical methods over deep learning.

The susceptibility of CNNs to pixel-level distortions was further highlighted by [38] and is in line with findings from [18], who demonstrated that noise disrupts the texture-based cues heavily relied upon by CNNs, leading to misclassification when visual details become unstable. These studies collectively emphasize the need to incorporate robustness-enhancing strategies during training, especially when models must operate on noisy or clinically degraded data. Motion blur led to a systematic decline in classifier performance, although the networks retained partial tolerance to small blur values. Under such conditions, CNNs were still able to recognize the general anatomical layout despite the loss of fine details. However, at high levels of blur, key morphological features became obscured, leading to a sharp increase in misclassifications. This effect aligns with prior observations [36,39,40], as well as blur-robustness analyses from [40,41], which consistently showed that blur weakens the discriminative features extracted by CNNs and creates discrepancies between human and machine perception. The present study also demonstrated that the deeper architecture (ResNet-34) achieved slightly better results than the shallower ResNet-18, suggesting that networks with a greater number of parameters possess a superior ability to generalize under conditions of partial loss of sharpness. Nonetheless, this difference was not substantial and did not offset the impact of severe blur. The effect of acoustic shadowing was of a different nature. Even narrow shadows caused noticeable deterioration in classification results, suggesting that networks interpreted the appearance of a dark region as a disturbance in feature extraction. However, further increases in shadow width did not result in a proportional decline in performance—the models partially adapted to the presence of uniform areas and exploited contextual information from the remaining visible regions. This phenomenon is consistent with studies on occlusion artifacts [42,43,44], as well as recent investigations by [28], who showed that occlusion disrupts CNN feature maps but can sometimes be compensated for by broader contextual patterns. Similar conclusions were reached by [44], who demonstrated that severe occlusion leads to a collapse of CNN feature representations and a marked decline in recognition accuracy, while human observers remain substantially more robust.

The Grad-CAM visualizations presented in Figure 16 provide additional insight into the mechanisms underlying these effects. Under baseline conditions, activation maps were concentrated in central cardiac regions corresponding to anatomically meaningful structures used for apical view recognition. After the introduction of simulated artifacts, the spatial distribution of model attention changed substantially. Motion blur reduced the prominence of anatomical boundaries, consistent with the loss of high-frequency structural information. Acoustic shadowing acted as a localized occlusion that obscured diagnostically relevant regions and forced the model to rely on incomplete contextual cues. In contrast, speckle noise affected the entire image and produced more diffuse activation patterns, suggesting increased sensitivity to noise-induced texture variations. These observations indicate that image degradations do not merely reduce classification confidence but can also alter the internal feature representations used by CNNs during view recognition.

In this study, a limited comparative analysis was also conducted using alternative architectures, including EfficientNet-B0 and a Vision Transformer model used as a frozen feature extractor combined with a logistic regression classifier. The obtained results were qualitatively consistent with those observed for the ResNet models, suggesting that the impact of image quality degradation on classification performance is largely independent of the specific model architecture. At the same time, it should be emphasized that these models were used solely for comparative purposes and in a limited manner, without full fine-tuning, due to the relatively small dataset size. According to the literature [13,14,31,32], both CNN-based architectures and Vision Transformers may exhibit limited generalization ability when trained on small medical datasets, due to the risk of overfitting and difficulties in learning representative image features. In particular, transformer-based models typically require substantially larger training datasets to effectively capture global dependencies.

Their findings reinforce the results of the present study by showing that dark, uniform regions–analogous to acoustic shadows in echocardiography–disrupt the continuity of local texture-based cues relied upon by CNNs and induce misclassification when critical spatial information is missing. Interpreting these findings requires reference to the literature on strategies for improving model robustness. Refs. [36,38] emphasized the importance of retraining with degraded images and incorporating architectural solutions to reduce sensitivity to noise. A broader perspective is offered by [45], who reviewed data augmentation and confirmed that introducing distortions during training substantially improves resilience to real-world artifacts. Similarly, frequency-based perspectives—such as the framework proposed by [46]—indicate that noise and blur distort feature distributions in the Fourier domain and that models explicitly leveraging frequency information generalize more effectively under degradation. In the context of echocardiography, the work of [21] is particularly relevant, demonstrating that TTE image degradation significantly reduces CNN accuracy and that contrastive training strategies can enhance robustness to speckle, blur, and acquisition-related distortions. More recently, ref. [40] demonstrated that including blurred images in the training set improves CNN resilience to distortions and enhances consistency with human perception. Such an approach appears particularly promising in echocardiography, where artifacts are inevitable and should be regarded as a natural component of the model’s training environment.

Beyond robustness-focused training, recent research highlights a broader technological shift toward multimodal and 3D-enhanced imaging frameworks, which offer new possibilities for overcoming the limitations identified in the present study. Modern cardiac AI pipelines increasingly integrate multimodal inputs, virtual 3D anatomical models, and mixed reality environments, enabling view classification systems to rely not only on the degraded 2D ultrasound frames but also on structural and spatial priors derived from complementary modalities. For instance, holographic mixed reality solutions have recently been used to support procedural planning in structural heart disease, demonstrating that interactive 3D anatomical reconstructions enhance spatial orientation and reduce ambiguity caused by incomplete or artifact-contaminated 2D views [47]. Likewise, contemporary overviews of cardiovascular medicine emphasize that multimodal data fusion—combining echocardiography, CT, MRI, and computational 3D modeling—has become central to next-generation diagnostic workflows, with direct implications for training AI models that are more resilient to noise, blur, and dropout artifacts [48]. Incorporating such multimodal and virtual modeling strategies may therefore represent a future direction for echocardiographic view classification, compensating for the vulnerabilities of CNNs observed under degraded imaging conditions.

The original contribution of the present study lies in the quantitative characterization of the effect of controlled image quality degradation on apical TTE view classification using an “artifact type × severity level → degradation curve” framework. In addition, by comparing deep architecture with a classical HOG + SVM baseline, the study showed that high performance under reference conditions does not automatically translate into greater robustness to artifacts. A particularly important finding was that severe speckle noise caused performance collapse across all evaluated methods, whereas motion blur and acoustic shadowing produced more differentiated degradation profiles that depended on image representation. These findings indicate that evaluating echocardiographic classifiers solely on reference-quality images is insufficient and should be complemented by a structured robustness analysis under degraded imaging conditions.

### 4.1. Summary Checklist of the Proposed Evaluation Framework

Given the growing use of automated echocardiographic view classification in downstream quantitative and diagnostic pipelines, performance assessment limited to aggregate accuracy metrics is insufficient. Clinically realistic image degradations may affect not only final classification decisions but also the stability and interpretability of model outputs. To provide a structured and transparent summary of the evaluation dimensions addressed in this study, Table 15 presents a checklist of key criteria used to assess the proposed classification framework.

### 4.2. Limitations of the Study

It should be emphasized that the present study was primarily exploratory and aimed to assess the impact of controlled image degradations on the classification performance of ResNet-18 and ResNet-34 architectures in transthoracic echocardiography. A major limitation is the relatively small size of the available dataset (Table 1), which restricts the ability to train deep neural networks and limits the reliability of broader generalizations. The learning curve analysis performed in this study further confirms that dataset size remains an important limiting factor for model performance, as balanced accuracy increased consistently with larger fractions of the training data (Figure 10). This suggests that further expansion of the dataset would likely lead to additional improvements in classification accuracy and model robustness. Although the dataset contained diagnostically high-quality recordings that supported stable model training, controlled data augmentation (rotations and horizontal flips) was used to increase spatial variability and partially mitigate the effects of limited sample size. Nevertheless, augmentation cannot replace true clinical diversity and therefore cannot fully compensate for the constraints imposed by a relatively small dataset.

An additional limitation is the restricted scope of the comparison across model families. Although the analysis was extended to include EfficientNet-B0, a simplified ViT + LR configuration, and the classical HOG + SVM baseline, this comparison does not constitute a full benchmark of systematically optimized architectures. In particular, the transformer-based model was used in a simplified configuration without full fine-tuning, whereas HOG + SVM served as a conservative reference method rather than as a competitively optimized classifier. Therefore, the comparative analysis should be interpreted as a contextual cross-family comparison rather than as an exhaustive architectural benchmark.

Another limitation is the class imbalance present in the dataset, particularly the predominance of the apical four-chamber (A4C) view. Such imbalance may bias model learning toward the most frequent category despite the use of class weighting during training. In addition, classification performance was evaluated at the frame level rather than at the level of entire video clips. While this approach enabled detailed analysis of degradation effects on individual images, it introduces statistical dependence between samples because frames originating from the same clip cannot be considered fully independent. The study also relied on simulated image degradations rather than naturally occurring clinical artifacts. Although the implemented motion blur, speckle noise, and acoustic shadowing resemble common disturbances observed in echocardiographic imaging, real artifacts often arise from more complex physical and anatomical interactions. For example, clinical speckle noise exhibits spatially correlated patterns dependent on tissue structure and ultrasound physics [41,42]. Similarly, acoustic shadowing in clinical imaging may vary depending on tissue composition and insonation angle, producing shadow morphologies that are more complex than those generated synthetically [43]. Motion artifacts observed in practice often result from non-linear patient movement or probe instability and may interact with other acoustic phenomena in ways that are difficult to reproduce using simple convolution-based blur kernels [15,34,44]. Previous studies have shown that synthetic ultrasound artifacts can approximate real distortions sufficiently for evaluating algorithmic robustness, although they cannot fully reproduce the complexity of real clinical imaging conditions [41,42]. Therefore, the present results should be interpreted as reflecting the behavior of CNN models under controlled degradation scenarios rather than as a direct representation of all real-world echocardiographic artifacts. Finally, the dataset was collected in a single clinical center using ultrasound equipment from one manufacturer. Consequently, the results may reflect center-specific acquisition protocols or device characteristics and should be validated in larger multi-center datasets acquired using different imaging systems.

## 5. Conclusions

The robustness of classification models to image quality degradation is a key prerequisite for the safe and reliable implementation of artificial intelligence in echocardiography. The conducted experiments showed that image degradation significantly reduces the accuracy of apical TTE view classification; however, the magnitude of this effect depends on artifact type. Speckle noise had the most destructive impact, leading to performance collapse across all evaluated methods at high severity. Motion blur and acoustic shadowing produced more differentiated degradation profiles that depended on the type of image representation. The additional comparative analysis with reference models further showed that high performance under reference conditions does not automatically translate into greater robustness to artifacts, and that model ranking may change under degradation.

These findings indicate that evaluating echocardiographic models solely on reference-quality images is insufficient and should be complemented by a structured robustness analysis under degraded imaging conditions. From a practical perspective, automated echocardiographic analysis systems should: (1) be trained on datasets that are as diverse as possible, including realistic artifacts or their simulations, (2) integrate image quality assessment and warning modules for reduced prediction reliability, and (3) be validated under extreme conditions in order to define the safe operating limits of the model. In this sense, this study contributes not only a quantitative description of how artifacts affect TTE view classification, but also a practical argument for including robustness testing in the standard evaluation of models used in echocardiography.

### Clinical Implications

The results obtained have direct practical significance. In real-world conditions, image artifacts—arising from a limited acoustic window, patient movement, or equipment parameters—are common. This means that AI models designed to support echocardiographic diagnostics must be tested and adapted to function with data deviating from ideal conditions. Without such adaptation, there is a risk of false classifications in clinical settings where images are noisy or partially obscured. In practice, this may lead to incorrect projection assignment and disrupt subsequent quantitative analyses (e.g., cardiac chamber volume measurements). Therefore, the development of solutions enabling automated image quality assessment and alerting in situations where classification reliability is compromised is crucial. Indeed, recent work demonstrated that a CNN-based pipeline can reliably perform real-time quality assessment of transthoracic echocardiogram frames, quantifying multiple quality indicators (e.g., anatomical visibility, clarity, depth-gain, foreshortening) and enabling rejection or alert of suboptimal images [49]. Introducing such mechanisms could enhance the clinical safety of AI applications in echocardiography. In parallel, enriching training processes with artifact-affected images is essential to ensure that models more accurately reflect clinical practice conditions and exhibit greater resilience to common disturbances.

## Figures and Tables

**Table 1:** Number of frames assigned to individual apical cardiac views in the training and test sets.

Projection	Frames in Training Set	Frames in Validation Set	Frames in Test Set
A2C	334	84	100
A3C	248	62	80
A4C	688	172	215
A5C	119	28	40

(A2C—apical two-chamber; A3C—apical three-chamber; A4C—apical four-chamber; A5C—apical five-chamber).

**Table 2:** Computational characteristics of the ResNet-18 and ResNet-34 architectures.

Model	Parameters [M]	FLOPs [G]	Inference Time [ms]
ResNet-18	11.18	4.74	22.08
ResNet-34	21.29	9.57	40.65

(M—10^6^; G—10^9^).

**Table 3:** Training configuration of the primary CNN models.

Parameter	Value
Architectures	ResNet-18, ResNet-34 (torchvision)
Pretraining	ImageNet-1K pretrained weights
Output head	Dropout (*p* = 0.3) + linear classification layer
Classes	A2C, A3C, A4C, A5C
Input resolution	256 × 256 RGB images
Normalization	ImageNet statistics
Data augmentation	random rotation (±10°), horizontal flip
Train/test split	sequence-level stratified 80/20
Validation split	frame-level stratified 20% of training data
Optimizer	AdamW
Learning rate	1 × 10^−4^ (backbone), 5 × 10^−4^ (classification head)
Weight decay	1 × 10^−4^
Loss function	cross-entropy with label smoothing (0.05)
Class imbalance handling	class-weighted loss + weighted sampling
Batch size	32
Maximum epochs	30
Learning-rate scheduler	cosine annealing
Early stopping	patience = 5 epochs

**Table 4:** Training configuration of additional models.

Model	Initialization	Training Strategy	Feature Representation	Classifier
EfficientNet-B0	ImageNet-1K pretrained	Full fine-tuning (same protocol as ResNet models)	Final feature maps	Linear layer
ViT-B/16	ImageNet-1K pretrained	Frozen backbone	CLS token embedding	Logistic Regression
HOG + SVM	HOG descriptor computed from grayscale images	no end-to-end training; handcrafted feature extraction	histograms of oriented gradients	Support Vector Machine

**Table 5:** Classification metrics (precision, recall, F1-score, AUC) for the ResNet-18 model under reference conditions.

Projection	Precision	Recall	F1-Score	AUC	Support
A2C	0.752 (0.666–0.836)	0.760 (0.676–0.842)	0.756 (0.687–0.824)	0.951 (0.931–0.969)	100
A3C	0.782 (0.691–0.871)	0.850 (0.772–0.928)	0.814 (0.744–0.878)	0.974 (0.959–0.985)	80
A4C	0.953 (0.923–0.979)	0.856 (0.809–0.899)	0.902 (0.869–0.93)	0.962 (0.942–0.979)	215
A5C	0.741 (0.625–0.843)	1.000 (1.0–1.0)	0.851 (0.769–0.914)	0.998 (0.994–0.999)	40
accuracy	0.846				435
balanced accuracy	0.866				

(A2C—apical two-chamber; A3C—apical three-chamber; A4C—apical four-chamber; A5C—apical five-chamber).

**Table 6:** Classification metrics (precision, recall, F1-score, AUC) for the ResNet-34 model under reference conditions.

Projection	Precision	Recall	F1-Score	AUC	Support
A2C	0.731 (0.648–0.809)	0.870 (0.802–0.933)	0.795 (0.728–0.848)	0.959 (0.941–0.974)	100
A3C	0.873 (0.794–0.945)	0.775 (0.682–0.869)	0.821 (0.748–0.887)	0.983 (0.972–0.991)	80
A4C	0.915 (0.876–0.949)	0.898 (0.856–0.936)	0.906 (0.877–0.931)	0.956 (0.934–0.975)	215
A5C	0.824 (0.687–0.942)	0.7 (0.549–0.837)	0.757 (0.631–0.857)	0.965 (0.942–0.982)	40
accuracy	0.851				435
balanced accuracy	0.81				

(A2C—apical two-chamber; A3C—apical three-chamber; A4C—apical four-chamber; A5C—apical five-chamber).

**Table 7:** Effect of motion blur intensity on Macro-AUC for the ResNet-18 model.

Motion Blur Kernel Size (k)	Baseline Macro-AUC	Macro-AUC Under Motion Blur	Δ AUC	95% CI for Δ	Bootstrap *p*-Value	Holm-Adjusted *p*-Value	Significant (α = 0.05)
0	0.981	0.981	0.000	[0.000, 0.000]	1.000	1.000	No
2.5		0.972	−0.009	[−0.027, 0.004]	0.186	0.372	No
5		0.947	−0.034	[−0.072, −0.005]	0.006	0.018	Yes
7.5		0.905	−0.076	[−0.143, −0.024]	0.000	0.000	Yes
10		0.876	−0.106	[−0.183, −0.038]	0.000	0.000	Yes
12.5		0.841	−0.140	[−0.237, −0.052]	0.000	0.000	Yes
15		0.813	−0.168	[−0.289, −0.061]	0.000	0.000	Yes
17.5		0.765	−0.216	[−0.344, −0.096]	0.000	0.000	Yes
20		0.757	−0.224	[−0.359, −0.099]	0.000	0.000	Yes

**Table 8:** Effect of motion blur intensity on Macro-AUC for the ResNet-34 model.

Motion Blur Kernel Size (k)	Baseline Macro-AUC	Macro-AUC Under Motion Blur	Δ AUC	95% CI for Δ	Bootstrap *p*-Value	Holm-Adjusted *p*-Value	Significant (α = 0.05)
0	0.976	0.976	0.000	[0.000, 0.000]	1.000	1.000	No
2.5		0.971	−0.005	[−0.021, 0.005]	0.506	1.000	No
5		0.968	−0.008	[−0.025, 0.003]	0.212	0.637	No
7.5		0.941	−0.035	[−0.080, −0.003]	0.016	0.065	No
10		0.926	−0.050	[−0.101, −0.009]	0.004	0.020	Yes
12.5		0.908	−0.068	[−0.132, −0.017]	0.000	0.000	Yes
15		0.895	−0.081	[−0.151, −0.021]	0.000	0.000	Yes
17.5		0.875	−0.101	[−0.171, −0.040]	0.000	0.000	Yes
20		0.864	−0.112	[−0.189, −0.047]	0.000	0.000	Yes

**Table 9:** Effect of acoustic shadow width on Macro-AUC for the ResNet-18 model.

Shadow Width Fraction	Baseline Macro-AUC	Macro-AUC Under Shadow	Δ AUC	95% CI for Δ	Bootstrap *p*-Value	Holm-Adjusted *p*-Value	Significant (α = 0.05)
0	0.981	0.981	0.000	[0.000, 0.000]	1.000	1.000	No
0.1		0.957	−0.025	[−0.053, 0.000]	0.057	0.113	No
0.2		0.932	−0.049	[−0.105, −0.008]	0.000	0.000	Yes
0.3		0.909	−0.073	[−0.141, −0.018]	0.000	0.000	Yes
0.4		0.858	−0.123	[−0.224, −0.037]	0.000	0.000	Yes
0.5		0.810	−0.171	[−0.276, −0.084]	0.000	0.000	Yes
0.6		0.801	−0.181	[−0.279, −0.094]	0.000	0.000	Yes
0.7		0.780	−0.201	[−0.306, −0.105]	0.000	0.000	Yes

**Table 10:** Effect of acoustic shadow width on Macro-AUC for the ResNet-34 model.

Shadow Width Fraction	Baseline Macro-AUC	Macro-AUC Under Shadow	Δ AUC	95% CI for Δ	Bootstrap *p*-Value	Holm-Adjusted *p*-Value	Significant (α = 0.05)
0	0.976	0.976	0.000	[0.000, 0.000]	1.000	1.000	No
0.1		0.951	−0.025	[−0.062, −0.003]	0.028	0.057	No
0.2		0.905	−0.071	[−0.139, −0.022]	0.000	0.000	Yes
0.3		0.914	−0.062	[−0.122, −0.019]	0.000	0.000	Yes
0.4		0.871	−0.105	[−0.184, −0.045]	0.000	0.000	Yes
0.5		0.828	−0.148	[−0.246, −0.069]	0.000	0.000	Yes
0.6		0.798	−0.178	[−0.286, −0.090]	0.000	0.000	Yes
0.7		0.847	−0.129	[−0.202, −0.068]	0.000	0.000	Yes

**Table 11:** Effect of speckle noise intensity on Macro-AUC for the ResNet-18 model.

Noise Amount	Baseline Macro-AUC	Macro-AUC Under Noise	Δ AUC	95% CI for Δ	Bootstrap *p*-Value	Holm-Adjusted *p*-Value	Significant (α = 0.05)
0	0.981	0.981	0.000	[0.000, 0.000]	1.000	1.000	No
0.5		0.832	−0.149	[−0.231, −0.077]	0.000	0.000	Yes
1		0.768	−0.213	[−0.306, −0.123]	0.000	0.000	Yes
1.5		0.706	−0.275	[−0.387, −0.165]	0.000	0.000	Yes
2		0.691	−0.290	[−0.411, −0.172]	0.000	0.000	Yes
2.5		0.684	−0.297	[−0.408, −0.184]	0.000	0.000	Yes
3		0.663	−0.319	[−0.445, −0.196]	0.000	0.000	Yes

**Table 12:** Effect of speckle noise intensity on Macro-AUC for the ResNet-34 model.

Noise Amount	Baseline Macro-AUC	Macro-AUC Under Noise	Δ AUC	95% CI for Δ	Bootstrap *p*-Value	Holm-Adjusted *p*-Value	Significant (α = 0.05)
0	0.976	0.976	0.000	[0.000, 0.000]	1.000	1.000	No
0.5		0.900	−0.076	[−0.131, −0.028]	0.000	0.000	Yes
1		0.690	−0.286	[−0.375, −0.187]	0.000	0.000	Yes
1.5		0.634	−0.342	[−0.443, −0.242]	0.000	0.000	Yes
2		0.588	−0.388	[−0.493, −0.295]	0.000	0.000	Yes
2.5		0.575	−0.401	[−0.515, −0.288]	0.000	0.000	Yes
3		0.591	−0.385	[−0.500, −0.261]	0.000	0.000	Yes

**Table 13:** Comparison of sequence-level balanced accuracy for the primary and reference models under reference conditions and under medium and high levels of simulated artifacts.

Model	Baseline	Motion Blur (10)	Motion Blur (20)	Acoustic Shadow (0.3)	Acoustic Shadow (0.7)	Speckle Noise (1.5)	Speckle Noise (3.0)
ResNet-18	0.885	0.482	0.426	0.607	0.320	0.358	0.250
ResNet-34	0.859	0.678	0.590	0.601	0.326	0.250	0.250
EfficientNet-B0	0.635	0.557	0.310	0.499	0.385	0.250	0.250
ViT + LR	0.668	0.256	0.300	0.324	0.313	0.368	0.270
HOG + SVM	0.531	0.553	0.533	0.490	0.562	0.261	0.261

**Table 14:** Representative failure cases illustrating systematic misclassification under image artifacts.

Model	Artifact Type	Intensity	True View	Predicted View	Δ True-Class Probability
ResNet-18	Motion blur	kernel = 5.0	A5C	A3C	−0.88
ResNet-18	Acoustic shadow	wf = 0.7	A5C	A2C	−0.65
ResNet-18	Speckle noise	Speckle = 3.0	A5C	A2C	−0.77
ResNet-34	Motion blur	kernel = 2.5	A2C	A3C	−0.89
ResNet-34	Acoustic shadow	Wf = 0.6	A2C	A3C	−0.75
ResNet-34	Speckle noise	Speckle =2.5	A3C	A2C	−0.89

Δ True-class probability denotes the difference between the probability assigned to the true class under baseline conditions and after artifact introduction.

**Table 15:** Checklist of evaluation criteria for the proposed echocardiographic view classification framework.

Criterion	Description	Addressed in This Study
Baseline performance	Classification accuracy and discrimination under reference (artifact-free) imaging conditions	Section 3.1, Figure 9
Robustness to artifacts	Performance degradation under controlled motion blur, acoustic shadowing, speckle noise, etc.	Section 3.2, Section 3.3, Section 3.4 and Section 3.5; Table 5, Table 6, Table 7, Table 8, Table 9, Table 10, Table 11 and Table 12
Probability stability	Changes in true-class probability distributions with increasing artifact severity	Section 3.7, Table 14
Architectural consistency	Comparison of degradation trends across ResNet-18 and ResNet-34	Section 3.2, Section 3.3, Section 3.4 and Section 3.5
Failure mode transparency	Qualitative analysis of systematic misclassifications under artifacts	Section 3.8

## Data Availability

Data supporting the reported results are available from the corresponding author on reasonable request.

## Abbreviations

The following abbreviations are used in this manuscript:

AI	artificial intelligence
CNN	Convolutional Neural Network
TTE	Transthoracic Echocardiography
A2C	Apical Two-Chamber
A3C	Apical Three-Chamber
A4C	Apical Four-Chamber
A5C	Apical Five-Chamber
FLOPs	Floating point operations per second
CPU	Central Processing Unit
CT	Computed Tomography
MRI	Magnetic Resonance Imaging
